# Genotyping by Sequencing-Based Discovery of SNP Markers and Construction of Linkage Map from F_5_ Population of Pepper with Contrasting Powdery Mildew Resistance Trait

**DOI:** 10.1155/2021/6673010

**Published:** 2021-03-15

**Authors:** Abinaya Manivannan, Sena Choi, Tae-Hwan Jun, Eun-Young Yang, Jin-Hee Kim, Eun-Su Lee, Hye-Eun Lee, Do-Sun Kim, Yul-Kyun Ahn

**Affiliations:** ^1^Vegetable Research Division, National Institute of Horticultural and Herbal Science, Rural Development Administration, Jeonju 55365, Republic of Korea; ^2^Department of Plant Bioscience, Pusan National University, Busan 46241, Republic of Korea; ^3^Department of Vegetable Crops, Korea National College of Agriculture and Fisheries, Jeonju 54874, Republic of Korea

## Abstract

Powdery mildew (PM) is a common fungal disease infecting pepper plants worldwide. Molecular breeding of pepper cultivars with powdery mildew resistance is desirable for the economic improvement of pepper cultivation. In the present study, 188 F_5_ population derived from AR1 (PM resistant) and TF68 (PM sensitive) parents were subjected to high-throughput genotyping by sequencing (GBS) for the identification of single nucleotide polymorphism (SNP) markers. Further, the identified SNP markers were utilized for the construction of genetic linkage map and QTL analysis. Overall read mapping percentage of 87.29% was achieved in this study with the total length of mapped region ranging from 2,956,730 to 25,537,525 bp. A total of 41,111 polymorphic SNPs were identified, and a final of 1,841 SNPs were filtered for the construction of a linkage map. A total of 12 linkage groups were constructed corresponding to each chromosome with 1,308 SNP markers with the map length of 2506.8 cM. Further, two QTLs such as *Pm-2.1* and *Pm-5.1* were identified in chromosomes 2 and 5, respectively, for the PM resistance. Overall, the outcomes of the present endeavor can be utilized for the marker-assisted selection of pepper with powdery mildew-resistant trait.

## 1. Introduction

Powdery mildew (PM) is a widely occurring disease in *Solanaceae* plants caused by an obligate fungus *Leveillula taurica* (Lev.) from the ascomycete family. The incidence of powdery mildew has been rising in both greenhouse and field grown pepper plants [1]. The primary symptom of premature defoliation caused by the fungus drastically reduces the plant growth, yield, and marketing of pepper plants. Moreover, the endophytic nature of *L. taurica* delimits the use of chemical control measures for disease prevention in an agricultural setting [2]. Therefore, the inevitable requirement of genetic resistance lines to powdery mildew arises. However, the traditional breeding methods for PM resistance may take upwards of 10 years. In order to provide a more rapid solution, the molecular marker-assisted breeding aided by modern sequencing technologies is evolving as the current breeding method of choice. Rapid innovations in genome sequencing platforms, such as next-generation sequencing (NGS), provide numerous opportunities for transcriptome assembly, functional annotation of genes, and identification of molecular markers [3–5]. New software tools in NGS technology enable the cost-effective identification, confirmation, and evaluation of genetic markers on a large scale. Among the NGS approaches, genotyping by sequencing (GBS) has been noted for its wide-range utilization for high-throughput analysis [6]. This process employs restriction enzyme-based complexity reduction coupled with DNA barcoded adapters to generate multiplex libraries of samples for NGS sequencing [7]. GBS has been demonstrated to be robust across a range of species and capable of producing large number of molecular markers which can be utilized for the construction of genetic maps [8]. GBS provides a rapid and low-cost tool for genotyping large populations, allowing breeders to implement genomic selection on a large scale in their breeding programs [9]. The GBS approach renders discovery of polymorphisms and simultaneously obtains the genotypic information across the whole population of interest. This synergistic approach makes GBS a promising and flexible platform for a wide range of species and germplasm sets.

From the past decade, DNA-based molecular markers are employed in plant breeding for genetic diversity and genome association analyses in several plants [10]. Major advancements in sequencing technology and bioinformatics methodologies prompted a transition from conventional genetics-based breeding to modern genomics-based marker-assisted breeding. Among the molecular markers, SNPs have been utilized for the genome-wide studies [11, 12]. NGS technologies have identified genome-wide SNPs in several crops, such as mung bean [13], barley [14], castor [15], cabbage [16], and grape [17]. One of the primary uses of DNA markers in molecular breeding is in the construction of linkage maps for diverse crop species. Molecular linkage maps and QTL mapping are valuable tools for characterizing the schematic view of loci associated with agronomically important quantitative traits like disease resistance. According to Qian et al. [18], the localization of resistance loci on linkage maps and identification linked of polymorphic DNA sequences greatly improve marker-assisted selection. Pepper is a widely consumed horticultural crop in *Solanaceae* which also includes other major vegetables such as potato, tomato, and eggplant. Peppers are used as vegetable, condiment, spice, medicine, coloring agent, and source of vitamins [4]. The most common cultivated pepper species are *Capsicum annuum*, *C. frutescens*, *C. chinense*, *C. pubescens*, and *C. baccatum* [19]. In pepper, several thousand SNP markers associated with various traits such as disease resistance [20], flowering [21], and pungency [22] have been discovered. Moreover, the genomics-based improvement of pepper has been enhanced after the release of pepper reference genome (*C. annuum* cv. CM334) with a genomic size of 3.48 Gb by Kim et al. [23]. The present study deals with the GBS-based discovery of SNP markers, construction of genetic linkage map, and QTL analysis from 188 F_5_ population obtained from pepper cultivars with contrasting powdery mildew resistance traits.

## 2. Materials and Methods

### 2.1. Plant Materials and Phenotypic Evaluation of Disease Resistance

Two cultivars of pepper AR1 (PM-resistant line) and TF68 (PM susceptible line) were grown in a greenhouse at the National Institute of Horticultural and Herbal Science, Rural Development Administration (RDA), Jeonju, Republic of Korea. The 188 F_5_ lines were produced by self-pollinating from F_1_ lines to F_4_ lines [24]. A total of 188 F_5_ population (single seed descendants) were selected for the present study. The plants were infected and scored according to Ahn et al. [19]; in detail, the plants were maintained in between the powdery mildew-affected plants in a polyvinyl house and grown for two weeks. After infection, the severity of the disease has been assessed from 0 to 4 scale (0: highly resistant, 1: resistant, 2: moderate, 3: sensitive, and 4: highly sensitive) (Supplementary Figure 1) based on the percentage of plants infected. The cultivars “Saengryeg 211” (PM sensitive) and “11PM37” (PM resistant) were used as controls.

### 2.2. Genotyping by Sequencing Analysis

The genomic DNA was isolated from the young leaves using the CTAB method according to our previous report [19]. The quantity and quality of DNA were assessed before the GBS library construction. A total of 188 F_5_ population were subjected to GBS analysis (SEEDERS, Daejeon, Korea). *Ape*K1 was employed for the genome reduction. Further, the digested fragments (approximately 100-400 bp) were sequenced using HiSeq 2000 system (Illumina, San Diego, CA, USA) according to Elshire et al. [9]. Demultiplexing has been performed using the barcode sequence, and adapter sequence removal and sequence quality trimming were performed. Adapter trimming was performed using cutadapt v. 1.8.3 [25], and sequence quality trimming has been performed using DynamicTrim and LengthSort of SolexaQA v.1.13 [26].

### 2.3. Discovery and Annotation of SNP Markers

The reads were aligned to the pepper reference genome using the Burrows-Wheeler Aligner (BWA 0.6.1-r104) program [27]. The default values for mapping have been used, except for seed length (−*l*) = 30, maximum differences in the seed (−*k*) = 1, number of threads (−*t*) = 16, maximum number of gap extensions (−*e*) = 50, mismatch penalty (−*M*) = 6, gap open penalty (−*O*) = 15, and gap extension penalty (−*E*) = 8. Mapped reads were extracted from the BAM file using SAMtools 0.1.16. An *in-house* script for the biallelic loci has been employed to select significant sites in the called SNP positions, and the SNP matrix was constructed by eliminating the miscalled SNP positions through SNP comparison among samples [28]. Further, the SNPs were classified into homozygous (SNP read depth ≥ 90%), heterozygous (40% ≤ SNP read depth ≤ 60%), and others (homozygous/heterozygous; could not be distinguished by type) based on their position [5]. The polymorphic SNPs between two samples with sufficient sequences on both sides of the SNP site, without structural variation, were noted adjacent to the SNP site. The SNPs were further filtered using the criteria of missing rate of <30% and minor allele frequency (>20%). The flanking sequences (600 bp) of the identified SNPs were used as the query for the Blastn-based homology search.

### 2.4. Linkage Map Construction and QTL Analysis

A single genetic map was developed from both parents using JoinMap 4.1 [29] with regression mapping algorithm. The population type “RI3” was employed. A minimum log likelihood (LOD) score of 8.0 was used, and the recombination rates were converted into the Kosambi mapping function to determine the map distance in centimorgans. The chi-square test (*p* < 0.001) was employed to eliminate the skewed SNPs, and the markers displaying identical segregation or more than five missing data points were filtered. MapChart 2.2 [30] was used for the visualization of the final genetic map. For the identification of QTLs, MapQTL 6.0 [31] was employed. The multiple QTL mapping (MQM) was performed to evaluate the association between the markers identified and the trait for powdery mildew disease resistance. The genome-wide threshold significance (LOD) was set to 4.5 with 1,000 permutation tests.

## 3. Results

### 3.1. Phenotypic Evaluation of Disease Resistance in 188 F_5_ Population

**Figure 1:**
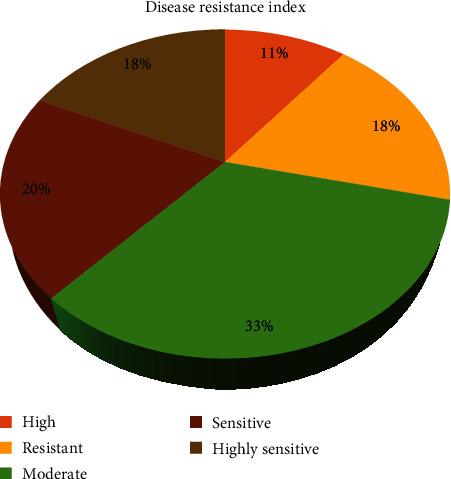
Phenotypic evaluation of powdery mildew disease resistance in 188 F_5_ population along with the parental lines of pepper.

A total of 188 F_5_ population were inoculated with the fungus, and the disease symptoms occurred after 20 days of inoculation. The disease index (DI) was classified in the scale of 0-4 denoting resistance to sensitive based on the percentage of plants affected (Figure 1). Among the 188 F_5_ population, the majority of the population displayed moderate DI with 33% followed by sensitive DI (20%). The resistant and highly resistant DI was observed in 18% and 11% of the population, respectively.

### 3.2. Genotyping by Sequencing (GBS) Analysis of 188 F_5_ Population

The overall summary of the GBS data is provided in Supplementary Table 1. In the parental lines, a total length of raw reads 2,289,576,676 bp has been obtained for AR1 which has been trimmed to 1,520,510.658 bp and the overall 1,557,295,366 bp length of raw reads was trimmed to 1,014,880,396 bp in TF68, respectively. Moreover, the total and the trimmed length of reads in the F_5_ population ranged from 49,469,194 bp to 2,328,589, 744 bp and 33,288,497 bp to 1,547,088,780 bp, respectively. The percentage of trimmed to raw reads of about 78.25 to 85.49% has been observed among the F_5_ population. Subsequently, the clean reads of each sample that have been demultiplexed and trimmed were mapped to the reference genome with the read mapping percentage of 69.89% and 89.16% for AR1 and TF68, respectively. However, the overall read mapping percentage of 87.29% has been acquired in this study with the total length of mapped region ranging from 2,956,730 to 25,537,525 bp. Among the F_5_ population, the overall reference genome coverage obtained is 0.534% with the range of 0.11 to 0.93%.

### 3.3. Discovery of SNP Markers

After the alignment of reads to the reference genome, the raw SNPs were detected and the SNP matrix has been generated between parental lines and 188 F_5_ population using raw SNPs of each sample (Supplementary Table 2). The average number of SNPs for each sample was 32,303; among them, 23,380 SNPs were homozygous and 3,369 SNPs were heterozygous types followed by 5,554 other types of SNPs. In detail, AR1 consisted of 81,223 total SNPs which comprised 75,784 homozygous SNPs, 1,664 heterozygous SNPs, and 3,775 other SNPs. Similarly, a total of 43,453 SNPs which can be further classified into homozygous (38,741), heterozygous (1,481), and others (3,231) have been identified in TF68. The total SNPs in the F_5_ population varied between 5,673 and 67,510 with the majority of homozygous SNPs followed by other types and heterozygous SNPs. From the SNP matrix constructed from the data obtained from 188 F_5_ population, a total of 529,145 SNP loci were identified which were further filtered to 41,111 polymorphic SNPs (minimum depth = 3), and after the elimination of SNPs using the missing data (<30%) and minor allele frequency (MAF > 20%) criteria, 1,841 SNPs remained as the final SNPs for the construction of a genetic map (Table 1). The details of the SNP position in reference genome along with the parental lines and genotype data constructed using the final filtered 1,841 SNPs for 188 population are provided in supplementary dataset 3. The genotype data suggested the clustering of SNPs with the TF68 parental line with 29.1% (b) and AR1 (a) with 20.5%and 17.3% of h type involving both parents.

### 3.4. Construction of Genetic Linkage Map and QTL Analysis

**Figure 2:**
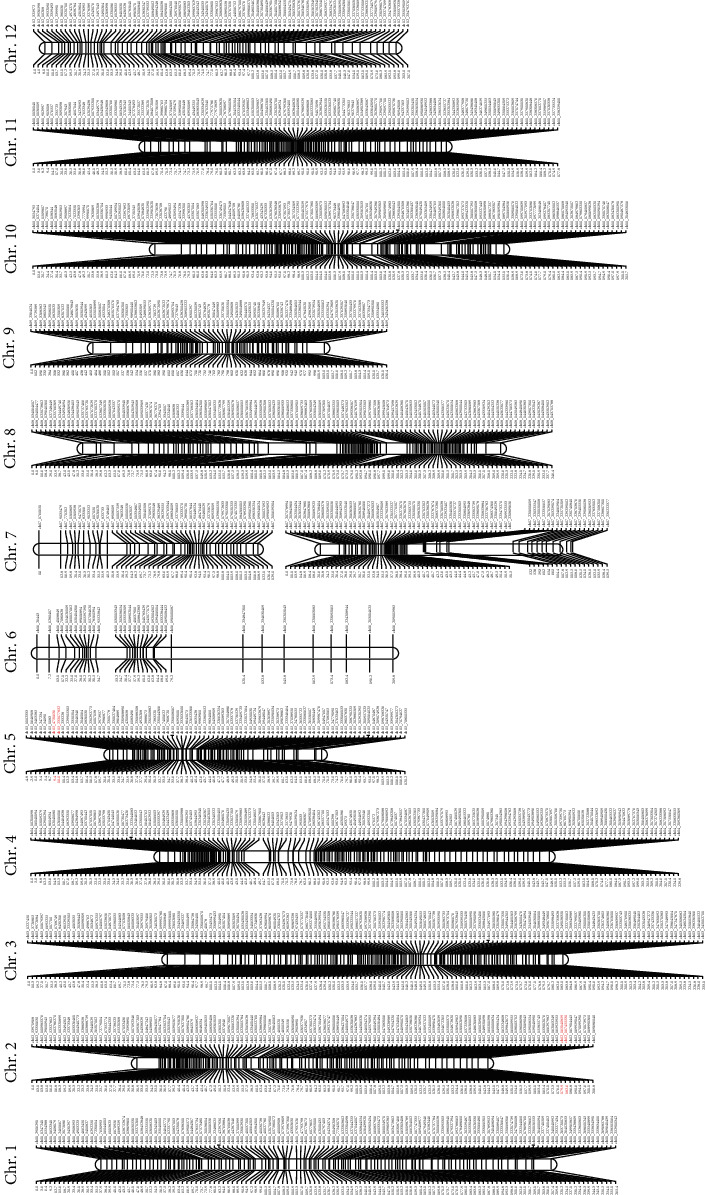
Linkage map containing the detailed loci of 1,308 SNP markers of AR1 and TF68 and the QTL flanking markers identified are marked in red color.

**Figure 3:**
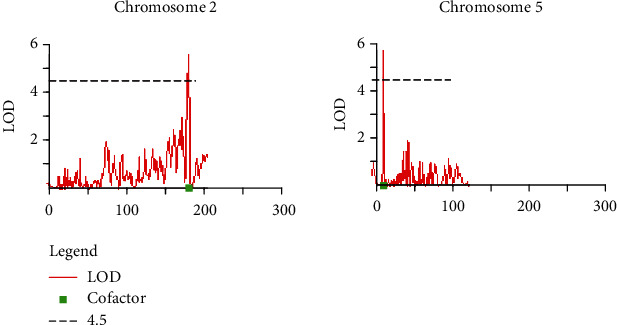
The LOD graph of two QTLs identified in chromosomes 2 and 5 using MQM analysis.

A total of 12 linkage groups were constructed with 1,308 SNP markers selected from the final filtered SNPs covering the total linkage map length of 2506.8 cM (Table 2, Figure 2). Among the linkage group, the highest number of SNP markers (150) has been mapped in the LG03 and the lowest number of markers has been noted in the LG06 (33). Moreover, the LG05 consisted of SNPs positioned with the lowest map length of 120.2 cM and LG01with the highest map length of 265.4 cM. In order to investigate the QTLs related to powdery mildew disease resistance, the genetic linkage map constructed was utilized along with the phenotype disease index data obtained for the 188 pepper population. The multiple QTL mapping approach revealed two significant QTLs for powdery mildew resistance such as Pm-2.1 and Pm-5.1 in 188 individuals of F_5_ population (Table 3). Both identified QTLs were minor QTLs with phenotypic variation (*R*^2^) less than 10% (Pm-2.1: 9.6% and Pm-5.1: 9.7%). The identified QTLs were mapped onto the genetic map of pepper (Figure 2). The LOD scores obtained for the identified QTLs were 5.55 and 5.64 for Pm-2.1 and Pm-5.1, respectively, which were greater than the LOD threshold (4.5) (Figure 3). The QTL Pm-2.1 was located between 179.9 and 182.6 cM and Pm-5.1 between 9.4 and 10.0 cM in chromosomes 2 and 5, respectively. Alleles conferring resistance to powdery mildew resistance were attributed by the resistant parent (“AR1”) because of the identified additive effects. Further, the annotation of the flanking SNP markers based on the homology search denoted that the flanking sequence corresponding to the SNP165140859-SNP167455530 position in chromosome 2 was identified as uncharacterized noncoding RNA. Further, the sequence similarity analysis of flanking marker identified in chromosome 5 (SNP4731636-SNP233077832) corresponded to the leucine-rich repeat (LRR) receptor-like serine/threonine-protein kinases.

## 4. Discussion

Advancements in the field of next-generation sequencing and its allied high-throughput technologies have revolutionized the discovery and genotyping of single nucleotide polymorphic markers in horticultural crops. In the present study, the genomes of 190 capsicum lines including the two parental cultivars with contrasting powdery mildew resistance traits have been reduced using the *ApekI* for GBS library construction. The *ApekI* restriction enzyme is selected due to its methylation sensitivity and the ability to excise the gene-rich area in the genome [32]. Further, the GBS library was constructed and sequenced using the Illumina HiSeq 2000 platform. The average of total number of raw reads (586,469,230 bp) in the present study has been higher than the reads reported by Pereira-Dias et al. [33]. Also, the percentage of trimmed to raw reads in the F_5_ population ranged between 78.25 and 85.49%. The trimmed reads were mapped to the reference genome with overall mapping percentage of 87.29% with 0.534% genome coverage. The GBS-generated sequence library was utilized for the discovery of SNP markers in the F_5_ population. According to Oh et al. [34], the GBS-based SNPs aided in the anchoring of high-resolution genetic map in *Pyrus pyrifolia* with high accuracy.

SNPs are considered as the marker of choice because of its wide range of advantages such as ease of automation for large-scale assays, accuracy, and diallelic nature. Due to the robustness of NGS technologies, several researchers have developed SNP markers from peppers [3, 4]. However, the SNPs identified in the current endeavor could render the vital marker sets for the breeding of powdery mildew-resistant varieties since the F_5_ population employed for the SNP identification has been derived from the interspecific breeding of sexually incompatible pepper species. In general, the progenies of interspecific breeding inherit economically important traits such as quality of fruits, resistance to diseases, and high composition of vital metabolites [35]. Similarly, the GBS-based SNP markers have been employed for the breeding of watermelon with *Fusarium* wilt resistance [36] and rubber trees [6] with resistance against fungal diseases. Moreover, the large number of SNPs discovered was majorly classified into homozygous type, illustrating that the sequence of reference genome could be produced from homozygous loci. Construction of the linkage map renders the initial basement for the QTL mapping. Previous studies have reported the construction of genetic map in pepper using both conventional methods and NGS-based methods in inter- and intraspecific capsicum varieties [37, 38]. However, the NGS-based mapping approach aids in the detection of large number of marker which facilitate the creation of high-density genetic maps. Recently, the high-resolution genetic map and QTL mapping of flowering traits have been constructed using the F_2_ population obtained from the interspecific cross between *C. annuum* and *C. chinense* [19].

In the present study, the linkage map was constructed using a total of 1,308 SNP markers distributed throughout the pepper genome. Similarly, a GBS-based identification of SNP markers was utilized for the construction of genetic linkage map and QTL identification for cucumber mosaic virus resistance in pepper plants [39]. In addition, the genetic linkage map was utilized for the identification of QTLs using the multiple QTL mapping approach. The MQM analysis produced two QTLs *Pm-2.1* and *Pm-5.1* in chromosomes 2 and 5, respectively. Previous reports have illustrated the utilization of MQM-based QTL markers in peppers [40, 41]. According to Eun et al. [39], the CMV resistance QTL has been identified in chromosomes 5 and 10. Recent study by Siddique et al. [42] has identified the QTLs associated with the *Phytophthora capsici* resistance in pepper. The report evidenced the presence of QTLs related to disease resistance in chromosomes 2 and 5. In addition, the presence of three major QTLs conferring broad spectrum resistance to *Phytophthora capsici* was mapped to chromosome 5 [42]. Similarly, the annotation of flanking SNP markers resulted in the identification of LRR receptor-like serine/threonine-protein kinases which have been widely studied for its disease resistance in pepper. Previous reports also suggested the identification of QTL regions flanked by the SNP markers in LRR-related genes rendering disease resistance in pepper against *Phytophthora capsici* [42].

Overall, the outcomes of the present study can be utilized for the development of molecular markers for the marker-assisted breeding of pepper with powdery mildew resistance.

## 5. Conclusions

The present study illustrated the genotyping by sequencing-based SNP marker discovery in 188 F_5_ pepper population derived from *C. annuum* with contrasting powdery mildew disease resistance characteristics. The disease resistance has been evaluated in all the lines, and the SNP markers identified have been annotated to understand the essential role in their associated genes. Moreover, SNPs identified have been utilized for the construction of the genetic linkage map. Further, the QTLs associated with the powdery mildew resistance have been identified. Overall, the outcomes of the present endeavor can be utilized for the development of molecular markers for the marker-assisted breeding of pepper with powdery mildew resistance.

## Figures and Tables

**Table 1:** Summary of the polymorphic SNP filtering in the F_5_ population of pepper plants along with the parental lines.

SNP details	Number of filtered SNPs
Total SNP loci	529,145
Polymorphic SNP (min. depth = 3)	41,111
Final filtered SNP markers (missing data < 30%; minor allele frequency > 20%)	1,841

**Table 2:** The SNP markers associated with the linkage group along with the map length.

Linkage group	No. of SNP marker on linkage map	Linkage map length (cM)
LG01	130	265.4
LG02	124	206.8
LG03	150	233.1
LG04	144	229.8
LG05	86	120.2
LG06	33	209.8
LG07a	48	129.0
LG07b	68	139.5
LG08	115	246.4
LG09	79	136.4
LG10	132	205.7
LG11	116	177.8
LG12	83	207.1
Total	1308	2506.8

**Table 3:** QTLs identified for the powdery mildew disease resistance with multiple QTL mapping analysis.

QTL	Chromosome	Flanking markers	QTL position (cM)	Additive effect	*R* ^2^ (%)	LOD score	LOD threshold
*Pm-2.1*	2	SNP165140859-SNP167455530	179.9-182.6	0.20	9.6	5.55	4.5
*Pm-5.1*	5	SNP4731636-SNP233077832	9.4-10.0	-0.25	9.7	5.64	4.5

## Data Availability

No data were used to support this study.

## Supplementary Materials

Supplementary Materials Supplementary Table 1: the complete GBS statistics of pepper population employed in the present study. Supplementary Table 2: the list of SNP markers discovered and classified into homozygous, heterozygous, and others in all the pepper population employed in this study. Supplementary data 3: the list of genotype data observed for the 188 population using the 1,841 final filtered SNP markers. Supplementary Figure 1: phenotype of the plants used for scoring the disease index in 0-4 scale.

## References

[1] Cerkauskas R. F., Ferguson G., Banik M. (2011). Powdery mildew (Leveillula taurica) on greenhouse and field peppers in Ontario–host range, cultivar response and disease management strategies. *Canadian Journal of Plant Pathology*.

[2] Elad Y., Messika Y., Brand M., David D. R., Sztejnberg A. (2007). Effect of microclimate on Leveillula taurica powdery mildew of sweet pepper. *Phytopathology*.

[3] Jo J., Venkatesh J., Han K. (2017). Molecular mapping of PMR1, a novel locus conferring resistance to powdery mildew in pepper (Capsicum annuum). *Frontiers in Plant Science*.

[4] Manivannan A., Kim J. H., Yang E. Y. (2018). Next-generation sequencing approaches in genome-wide discovery of single nucleotide polymorphism markers associated with pungency and disease resistance in pepper. *BioMed Research International*.

[5] Ryu J., Kim W. J., Im J. (2019). Single nucleotide polymorphism (SNP) discovery through genotyping-by- sequencing (GBS) and genetic characterization of Dendrobium mutants and cultivars. *Scientia Horticulturae*.

[6] Pootakham W., Ruang-Areerate P., Jomchai N. (2015). Construction of a high-density integrated genetic linkage map of rubber tree (Hevea brasiliensis) using genotyping-by-sequencing (GBS). *Frontiers in Plant Science*.

[7] Chung Y. S., Choi S. C., Jun T. H., Kim C. (2017). Genotyping-by-sequencing: a promising tool for plant genetics research and breeding. *Horticulture, Environment, and Biotechnology*.

[8] Deschamps S., Llaca V., May G. D. (2012). Genotyping-by-sequencing in plants. *Biology*.

[9] Elshire R. J., Glaubitz J. C., Sun Q. (2011). A robust, simple genotyping-by-sequencing (GBS) approach for high diversity species. *PLoS One*.

[10] Nadeem M. A., Nawaz M. A., Shahid M. Q. (2018). DNA molecular markers in plant breeding: current status and recent advancements in genomic selection and genome editing. *Biotechnology & Biotechnological Equipment*.

[11] Prom L. K., Ahn E., Isakeit T., Magill C. (2019). GWAS analysis of sorghum association panel lines identifies SNPs associated with disease response to Texas isolates of Colletotrichum sublineola. *Theoretical and Applied Genetics*.

[12] Chong X., Su J., Wang F. (2019). Identification of favorable SNP alleles and candidate genes responsible for inflorescence-related traits via GWAS in chrysanthemum. *Plant Molecular Biology*.

[13] Van K., Kang Y. J., Han K. S. (2013). Genome-wide SNP discovery in mungbean by Illumina HiSeq. *Theoretical and Applied Genetics*.

[14] Poland J. A., Brown P. J., Sorrells M. E., Jannink J. L. (2012). Development of high-density genetic maps for barley and wheat using a novel two-enzyme genotyping-by-sequencing approach. *PLoS One*.

[15] Senthilvel S., Ghosh A., Shaik M., Shaw R. K., Bagali P. G. (2019). Development and validation of an SNP genotyping array and construction of a high-density linkage map in castor. *Scientific Reports*.

[16] Xu Y., Zeng A., Song L., Li J., Yan J. (2019). Comparative transcriptomics analysis uncovers alternative splicing events and molecular markers in cabbage (Brassica oleracea L.). *Planta*.

[17] Guo D. L., Zhao H. L., Li Q. (2019). Genome-wide association study of berry-related traits in grape [ _Vitis vinifera_ L.] based on genotyping-by-sequencing markers. *Horticulture research*.

[18] Qian Y.-l., Wang X.-s., Wang D.-z. (2013). The detection of QTLs controlling bacterial wilt resistance in tobacco (N. tabacum L.). *Euphytica*.

[19] Ahn Y. K., Manivannan A., Karna S. (2018). Whole genome resequencing of Capsicum baccatum and Capsicum annuum to discover single nucleotide polymorphism related to powdery mildew resistance. *Scientific Reports*.

[20] Ahn Y.-K., Karna S., Jun T.-H. (2016). Complete genome sequencing and analysis of Capsicum annuum varieties. *Molecular Breeding*.

[21] Zhu Z., Sun B., Wei J. (2019). Construction of a high density genetic map of an interspecific cross of Capsicum chinense and Capsicum annuum and QTL analysis of floral traits. *Scientific Reports*.

[22] Garcés-Claver A., Fellman S. M., Gil-Ortega R., Jahn M., Arnedo-Andrés M. S. (2007). Identification, validation and survey of a single nucleotide polymorphism (SNP) associated with pungency in Capsicum spp. *Theoretical and Applied Genetics*.

[23] Kim S., Park M., Yeom S. I. (2014). Genome sequence of the hot pepper provides insights into the evolution of pungency in Capsicum species. *Nature Genetics*.

[24] Yoon J. B., Yang D. C., Do J. W., Park H. G. (2006). Overcoming two post-fertilization genetic barriers in interspecific hybridization between Capsicum annuum and C. baccatum for introgression of Anthracnose resistance. *Breeding Science*.

[25] Martin M. (2011). Cutadapt removes adapter sequences from high-throughput sequencing reads. *EMBnet Journal*.

[26] Cox M. P., Peterson D. A., Biggs P. J. (2010). SolexaQA: at-a-glance quality assessment of Illumina second-generation sequencing data. *BMC bioinformatics*.

[27] Li H., Durbin R. (2010). Fast and accurate long-read alignment with Burrows-Wheeler transform. *Bioinformatics*.

[28] Kim J. E., Oh S. K., Lee J. H., Lee B. M., Jo S. H. (2014). Genome-wide SNP calling using next generation sequencing data in tomato. *Molecules and Cells*.

[29] Van Ooijen J. W. (2006). *JoinMap® 4.1, software for calculation of genetic linkage maps in experimental populations; Kyazma BV*.

[30] Voorrips R. E. (2006). *Mapchart 2.2*.

[31] Ooijen J., Kyazma B. (2009). *MapQTL 6.0*.

[32] Taranto F., D’Agostino N., Grew B., Cardi T., Tripodi P. (2016). Genome-wide SNP discovery and population structure analysis in pepper (Capsicum annuum) using genotyping by sequencing. *BMC Genomics*.

[33] Pereria-Dias L., Vilanova S., Fita A., Prohens J., Rodriguez-Burruezo A. (2019). Genetic diversity, population structure, and relationship in a collection of pepper (Capsicum spp.) landraces from the Spanish centre of diversity revealed by genotyping-by-sequencing (GBS). *Horticulture Research.*.

[34] Oh S., Oh Y., Kim K. (2020). Construction of high-resolution genetic linkage map in pear pseudo-BC1 ((Pyrus pyrifolia × P. communis) × P. pyrifolia) using GBS-SNPs and SSRs. *Horticulture, Environment, and Biotechnology*.

[35] Rodríguez-Burruezo A., Prohens J., Raigón M. D., Nuez F. (2009). Variation for bioactive compounds in ají (Capsicum baccatum L.) and rocoto (C. pubescens R. & P.) and implications for breeding. *Euphytica*.

[36] Meru G., McGregor C. (2016). Genotyping by sequencing for SNP discovery and genetic mapping of resistance to race 1 of Fusarium oxysporum in watermelon. *Scientia Horticulturae*.

[37] Kang B. C., Nahm S. H., Huh J. H. (2001). An interspecific (Capsicum annuum ×C. chinese) F_2_ linkage map in pepper using RFLP and AFLP markers. *Theoretical and Applied Genetics*.

[38] Yarnes S. C., Ashrafi H., Reyes-Chin-Wo S., Hill T. A., Stoffel K. M., Van Deynze A. (2012). Identification of QTLs for capsaicinoids, fruit quality, and plant architecture-related traits in an interspecific Capsicum RIL population. *Genome*.

[39] Eun M. H., Han J. H., Yoon J. B., Lee J. (2016). QTL mapping of resistance to the cucumber mosaic virus P1 strain in pepper using a genotyping-by-sequencing analysis. *Horticulture, Environment, and Biotechnology*.

[40] Saeko N., Raweerotwiboon A., Petchaboon K., Tongyoo P., Srisawad N., Chunwongse J. (2020). QTL mapping of yield related components in double haploid (DH) pepper population derived from F1 of ‘PEPAC7’×‘PEPAC92’ cultivars. *Agricultural Science Journal*.

[41] Pongjareankit S., Saeko A., Lithanatudom S. K., Struss D. (2020). QTL mapping specific to Thrips palmi resistance in Capsicum annuum. *Asia-Pacific Journal of Science and Technology*.

[42] Siddique M. I., Lee H. Y., Ro N. Y. (2019). Identifying candidate genes for _Phytophthora capsici_ resistance in pepper ( _Capsicum annuum_ ) via genotyping-by-sequencing-based QTL mapping and genome-wide association study. *Scientific Reports*.

