# Supplementary material for: Genotyping by Sequencing-Based Discovery of SNP Markers and Construction of Linkage Map from F5 Population of Pepper with Contrasting Powdery Mildew Resistance Trait
Source: Biomed Res Int. 2021 Mar 15;2021:6673010. doi: 10.1155/2021/6673010 (PMC7987414; doi:10.1155/2021/6673010)
Supplement: Supplementary Materials — Supplementary Table 1: the complete GBS statistics of pepper population employed in the present study. Supplementary Table 2: the list of SNP markers discovered and classified into homozygous, heterozygous, and others in all the pepper population employed in this study. Supplementary data 3: the list of genotype data observed for the 188 population using the 1,841 final filtered SNP markers. Supplementary Figure 1: phenotype of the plants used for scoring the disease index in 0-4 scale. [file 6673010.f1.zip › 6673010.f1/Supplementary Table 1.pdf]

Supplementary Table 1. Summary of GBS sequencing in 188 F_5 population and two parental cultivars.

Sample	Total length of raw reads	Total length of trimmed reads (bp)	Avg. length of trimmed reads (bp)	Sum of trimmed reads	No. of mapped reads	[z] Percent of mapped reads (%)	No. of mapped region	Total length of mapped region (bp)	Avg. length of mapped region (bp)	Reference Genome coverage (%)
AR1	2,289,576,676	1,520,510,658	81.47	18658590	13,039,950	69.89%	137,409	19,415,752	141.30	0.7051%
TF68	1,557,295,366	1,014,880,396	81.05	12584922	11,220,700	89.16%	145,876	21,695,452	148.73	0.7879%
1.	568,617,274	392,307,925	82.88	4733280	4,192,922	88.58%	110,413	16,351,012	148.09	0.5938%
2.	344,987,518	238,929,335	83.10	2875084	2,515,737	87.50%	88,714	13,157,842	148.32	0.4779%
3.	677,382,962	470,643,765	82.80	5684254	4,931,317	86.75%	121,356	18,054,259	148.77	0.6557%
4.	981,281,458	680,582,183	83.06	8193836	7,226,289	88.19%	134,842	20,158,610	149.50	0.7321%
5.	544,718,250	376,490,587	83.08	4531564	3,938,238	86.91%	111,167	16,459,328	148.06	0.5978%
6.	1,031,075,872	713,829,178	82.98	8602412	7,553,381	87.81%	138,280	20,707,013	149.75	0.7520%
7.	614,812,250	426,408,209	82.24	5184988	4,539,516	87.55%	117,804	17,750,797	150.68	0.6447%
8.	557,067,722	386,100,899	82.84	4661064	4,114,746	88.28%	109,138	16,510,295	151.28	0.5996%
9.	628,429,474	432,054,347	82.68	5225406	4,543,229	86.94%	111,625	16,558,386	148.34	0.6014%
10.	708,650,340	484,195,667	82.18	5891850	5,206,951	88.38%	115,372	17,035,194	147.65	0.6187%
11.	798,524,584	540,954,494	82.47	6559022	5,756,322	87.76%	120,742	17,832,453	147.69	0.6476%
12.	879,223,180	597,610,892	82.16	7273330	6,328,060	87.00%	127,757	18,941,438	148.26	0.6879%
13.	1,295,786,368	883,172,263	82.38	10720178	9,146,082	85.32%	150,281	22,282,747	148.27	0.8093%
14.	742,815,812	515,545,550	82.76	6229722	5,408,907	86.82%	126,025	18,673,080	148.17	0.6782%
15.	798,488,628	534,043,406	81.43	6558598	5,744,440	87.59%	125,400	18,583,520	148.19	0.6749%
16.	1,721,944,152	1,177,571,203	81.92	14374966	12,497,206	86.94%	168,629	25,425,546	150.78	0.9234%
17.	342,002,766	232,373,671	82.16	2828306	2,474,833	87.50%	90,504	13,355,795	147.57	0.4850%
18.	77,464,778	51,764,409	82.39	628308	541,662	86.21%	30,145	4,042,612	134.11	0.1468%
19.	1,174,547,180	808,534,221	82.66	9781060	8,625,682	88.19%	146,176	22,010,850	150.58	0.7994%
20.	402,414,098	272,910,204	82.46	3309556	2,921,157	88.26%	97,969	14,408,549	147.07	0.5233%
21.	465,725,140	318,556,398	82.51	3861012	3,370,263	87.29%	108,778	16,337,342	150.19	0.5933%
22.	140,426,360	96,922,278	82.00	1181964	1,026,850	86.88%	48,982	6,903,047	140.93	0.2507%
23.	236,218,598	159,642,656	81.29	1963932	1,727,814	87.98%	71,408	10,369,032	145.21	0.3766%
24.	675,697,272	464,239,762	82.71	5612864	4,904,125	87.37%	123,802	18,387,328	148.52	0.6678%
25.	1,401,888,080	952,976,874	82.76	11515036	10,148,497	88.13%	155,587	23,134,578	148.69	0.8402%
26.	786,601,130	551,315,419	82.81	6657824	5,858,852	88.00%	130,573	19,614,246	150.22	0.7123%
27.	1,880,167,116	1,302,303,517	82.72	15743454	13,784,440	87.56%	165,943	24,642,430	148.50	0.8949%
28.	826,963,558	572,584,543	82.56	6935506	5,988,732	86.35%	142,644	21,681,132	151.99	0.7874%
29.	168,182,170	113,306,272	81.72	1386578	1,227,868	88.55%	56,762	8,095,603	142.62	0.2940%
30.	258,155,798	175,503,849	82.21	2134730	1,834,636	85.94%	75,885	10,973,040	144.60	0.3985%
31.	1,712,294,612	1,173,410,618	82.44	14232872	12,363,518	86.87%	173,993	25,537,525	146.77	0.9275%
32.	121,469,064	82,147,321	81.79	1004384	881,990	87.81%	44,254	6,221,498	140.59	0.2259%
33.	454,146,702	311,006,610	82.45	3772018	3,294,018	87.33%	100,937	14,936,454	147.98	0.5425%
34.	751,344,252	506,205,988	81.27	6229010	5,451,793	87.52%	117,089	17,227,730	147.13	0.6257%
35.	784,476,292	539,072,848	81.73	6595820	5,750,656	87.19%	124,297	18,541,438	149.17	0.6734%

36.	401,017,874	269,811,913	81.92	3293528	2,854,840	86.68%	94,300	13,764,558	145.97	0.4999%
37.	987,533,964	673,095,760	82.44	8164708	7,066,373	86.55%	125,761	18,685,583	148.58	0.6786%
38.	300,142,508	200,656,544	81.67	2457034	2,145,289	87.31%	80,538	11,664,547	144.83	0.4236%
39.	557,480,004	385,046,383	82.33	4676740	4,111,790	87.92%	113,085	16,764,792	148.25	0.6089%
40.	622,435,326	421,572,846	81.22	5190412	4,595,881	88.55%	114,965	17,132,340	149.02	0.6222%
41.	231,566,538	158,029,085	81.51	1938830	1,706,978	88.04%	69,978	10,302,536	147.23	0.3742%
42.	356,266,188	242,514,548	82.04	2956070	2,594,933	87.78%	89,230	12,965,851	145.31	0.4709%
43.	378,583,350	254,589,299	80.84	3149344	2,773,892	88.08%	93,905	13,878,236	147.79	0.5040%
44.	102,318,656	68,932,020	81.53	845530	735,422	86.98%	36,347	4,973,850	136.84	0.1806%
45.	643,190,422	438,990,857	82.35	5330762	4,687,970	87.94%	117,066	17,063,063	145.76	0.6197%
46.	678,145,310	457,950,005	82.13	5575962	4,920,814	88.25%	119,352	18,049,249	151.23	0.6555%
47.	80,173,800	53,732,051	81.90	656032	575,160	87.67%	32,402	4,375,821	135.05	0.1589%
48.	524,664,296	356,841,842	82.17	4342642	3,816,260	87.88%	102,466	15,079,350	147.16	0.5476%
49.	136,662,292	94,239,602	82.11	1147700	1,000,901	87.21%	47,156	6,751,309	143.17	0.2452%
50.	119,295,544	80,990,630	81.94	988408	871,263	88.15%	43,523	6,056,079	139.15	0.2199%
51.	361,721,804	246,500,666	81.85	3011790	2,604,580	86.48%	87,525	12,891,290	147.29	0.4682%
52.	654,347,690	446,696,101	81.88	5455760	4,806,404	88.10%	109,819	15,883,576	144.63	0.5769%
53.	216,329,880	145,323,157	81.66	1779656	1,568,169	88.12%	69,760	10,101,592	144.80	0.3669%
54.	1,809,050,996	1,099,017,215	78.42	14014890	12,222,607	87.21%	167,657	24,831,959	148.11	0.9018%
55.	200,039,994	132,651,622	81.18	1634084	1,410,078	86.29%	58,845	8,411,015	142.94	0.3055%
56.	401,962,224	271,048,379	80.96	3347990	2,940,649	87.83%	98,885	14,727,317	148.93	0.5349%
57.	632,774,090	435,878,915	81.42	5353140	4,712,869	88.04%	120,975	18,127,250	149.84	0.6583%
58.	806,831,228	551,974,170	81.90	6739910	5,935,511	88.07%	127,458	18,966,442	148.81	0.6888%
59.	662,487,078	450,929,112	81.58	5527602	4,822,462	87.24%	123,623	18,612,716	150.56	0.6760%
60.	119,081,424	80,939,002	81.67	991032	870,822	87.87%	46,249	6,333,493	136.94	0.2300%
61.	748,616,040	516,553,618	81.77	6317356	5,541,410	87.72%	129,926	19,387,769	149.22	0.7041%
62.	274,713,738	184,650,662	81.20	2274058	2,008,857	88.34%	78,837	11,513,735	146.04	0.4181%
63.	564,424,764	378,458,690	80.92	4677050	4,118,211	88.05%	111,409	16,597,501	148.98	0.6028%
64.	500,222,700	338,964,776	81.60	4153760	3,591,542	86.46%	102,906	15,208,212	147.79	0.5523%
65.	215,221,506	139,715,668	79.61	1755034	1,549,734	88.30%	70,526	10,279,556	145.76	0.3733%
66.	165,862,806	110,441,647	80.33	1374896	1,208,968	87.93%	59,045	8,420,098	142.60	0.3058%
67.	60,748,066	41,165,277	81.79	503306	443,820	88.18%	25,353	3,305,249	130.37	0.1200%
68.	183,971,904	123,447,032	81.73	1510486	1,331,101	88.12%	63,472	9,018,939	142.09	0.3275%
69.	120,642,076	81,047,388	82.00	988424	868,661	87.88%	46,691	6,439,001	137.91	0.2338%
70.	733,288,684	493,112,552	81.57	6044948	5,111,520	84.56%	120,505	17,835,476	148.01	0.6477%
71.	228,871,252	155,346,419	81.93	1896038	1,664,091	87.77%	73,484	10,619,636	144.52	0.3857%
72.	234,858,734	158,026,809	81.01	1950716	1,709,142	87.62%	70,477	10,368,971	147.13	0.3766%
73.	253,020,756	171,513,113	81.58	2102374	1,826,972	86.90%	70,152	10,256,589	146.21	0.3725%
74.	238,764,404	158,783,231	81.14	1956910	1,701,528	86.95%	71,110	10,352,876	145.59	0.3760%
75.	244,719,970	162,827,946	81.17	2006134	1,761,899	87.83%	71,430	10,618,847	148.66	0.3856%
76.	400,013,328	270,329,082	81.42	3320032	2,859,065	86.12%	95,576	14,010,400	146.59	0.5088%
77.	74,854,534	50,064,717	80.73	620122	540,224	87.12%	31,985	4,198,606	131.27	0.1525%
78.	340,588,362	227,424,638	80.56	2822900	2,445,457	86.63%	87,080	12,727,740	146.16	0.4622%
79.	225,298,680	151,271,296	81.23	1862246	1,632,848	87.68%	69,242	10,146,508	146.54	0.3685%
80.	397,808,296	265,748,280	80.95	3282748	2,836,038	86.39%	99,849	14,718,832	147.41	0.5345%

81.	779,574,156	519,453,517	80.75	6432584	5,655,052	87.91%	124,934	18,444,581	147.63	0.6699%
82.	49,469,194	33,288,497	81.75	407176	358,906	88.15%	23,227	2,956,730	127.30	0.1074%
83.	73,864,936	49,119,411	81.25	604522	523,108	86.53%	30,160	3,956,461	131.18	0.1437%
84.	194,921,516	131,786,923	81.25	1621920	1,412,345	87.08%	63,326	9,014,249	142.35	0.3274%
85.	333,321,008	227,023,292	81.61	2781742	2,430,790	87.38%	90,968	13,216,500	145.29	0.4800%
86.	157,298,410	106,041,182	81.58	1299898	1,124,198	86.48%	53,494	7,342,049	137.25	0.2666%
87.	557,893,498	376,920,020	81.65	4616416	4,062,444	88.00%	110,113	16,549,627	150.30	0.6010%
88.	521,552,082	349,512,254	81.40	4293912	3,704,674	86.28%	111,206	16,642,430	149.65	0.6044%
89.	1,172,928,554	784,837,615	81.60	9618628	8,470,589	88.06%	148,175	22,003,424	148.50	0.7991%
90.	526,790,144	353,898,875	81.35	4350300	3,779,903	86.89%	111,858	16,953,066	151.56	0.6157%
91.	687,066,842	469,192,918	81.28	5772616	4,918,772	85.21%	132,309	19,895,542	150.37	0.7226%
92.	688,679,004	467,895,245	81.25	5759000	4,973,470	86.36%	124,424	18,464,024	148.40	0.6706%
93.	825,716,208	555,488,408	80.75	6879330	5,834,818	84.82%	130,758	19,491,818	149.07	0.7079%
94.	436,246,068	288,009,277	80.45	3580160	3,125,434	87.30%	94,746	14,004,432	147.81	0.5086%
95.	250,834,106	162,365,289	80.24	2023460	1,771,980	87.57%	64,594	10,199,292	157.90	0.3704%
96.	194,471,662	126,610,014	80.28	1577192	1,360,939	86.29%	56,384	8,777,406	155.67	0.3188%
97.	486,292,780	319,586,038	80.34	3977990	3,470,339	87.24%	93,954	14,897,457	158.56	0.5410%
98.	528,862,058	344,160,548	80.31	4285246	3,762,587	87.80%	95,693	15,245,190	159.31	0.5537%
99.	1,843,398,066	1,211,692,187	80.91	14976476	13,184,208	88.03%	150,535	22,353,043	148.49	0.8118%
100.	487,461,552	317,175,785	80.38	3945924	3,379,196	85.64%	93,745	14,518,777	154.88	0.5273%
101.	533,589,262	348,223,730	79.75	4366626	3,833,847	87.80%	100,392	15,664,667	156.04	0.5689%
102.	472,607,684	309,650,210	80.15	3863402	3,414,555	88.38%	93,710	14,733,937	157.23	0.5351%
103.	352,541,106	228,408,912	80.10	2851440	2,484,631	87.14%	78,942	12,455,097	157.78	0.4523%
104.	258,109,944	165,917,836	79.60	2084288	1,831,637	87.88%	66,478	10,346,847	155.64	0.3758%
105.	798,138,764	513,192,023	80.02	6413402	5,597,796	87.28%	109,211	16,695,190	152.87	0.6063%
106.	486,089,366	313,009,990	79.85	3919884	3,417,998	87.20%	89,187	14,064,041	157.69	0.5108%
107.	536,514,626	345,568,993	79.89	4325292	3,791,003	87.65%	90,647	14,088,250	155.42	0.5116%
108.	762,272,250	498,949,465	80.41	6204812	5,416,059	87.29%	110,880	17,217,457	155.28	0.6253%
109.	742,352,828	469,944,931	79.14	5937922	5,139,912	86.56%	105,850	16,497,010	155.85	0.5991%
110.	780,098,144	501,488,005	79.28	6325914	5,511,002	87.12%	108,014	16,420,766	152.02	0.5964%
111.	911,942,332	589,468,347	79.76	7390758	6,445,802	87.21%	119,511	18,204,804	152.33	0.6612%
112.	482,345,296	308,731,826	79.96	3860852	3,420,412	88.59%	93,861	14,236,662	151.68	0.5170%
113.	502,436,620	325,035,950	79.42	4092496	3,579,850	87.47%	86,784	13,221,872	152.35	0.4802%
114.	657,723,312	423,388,484	79.82	5304582	4,673,296	88.10%	108,866	16,403,822	150.68	0.5957%
115.	1,091,675,266	708,160,252	80.01	8851138	7,763,662	87.71%	122,350	18,568,897	151.77	0.6744%
116.	2,328,589,744	1,547,088,780	80.28	19270820	16,816,257	87.26%	168,587	24,953,886	148.02	0.9063%
117.	667,329,422	430,349,162	79.10	5440516	4,767,704	87.63%	107,809	16,356,740	151.72	0.5940%
118.	866,239,832	561,172,518	79.97	7017014	6,092,794	86.83%	105,300	15,859,251	150.61	0.5760%
119.	980,071,276	626,000,730	80.05	7820332	6,816,671	87.17%	126,212	19,805,605	156.92	0.7193%
120.	217,177,876	142,829,700	79.92	1787166	1,564,416	87.54%	63,460	9,989,340	157.41	0.3628%
121.	520,253,626	337,317,764	79.83	4225628	3,736,524	88.43%	100,389	15,814,489	157.53	0.5743%
122.	430,749,850	280,782,470	80.00	3509706	3,059,847	87.18%	88,530	13,680,796	154.53	0.4969%
123.	728,249,592	468,958,267	79.75	5880668	5,166,289	87.85%	113,330	17,421,343	153.72	0.6327%
124.	688,945,240	444,315,952	80.19	5540636	4,830,408	87.18%	101,844	15,800,018	155.14	0.5738%
125.	552,812,592	352,741,842	79.55	4434108	3,595,979	81.10%	97,705	15,294,124	156.53	0.5554%

126.	1,022,116,970	655,714,830	79.42	8256800	7,175,853	86.91%	130,324	19,790,054	151.85	0.7187%
127.	893,938,880	574,656,389	79.76	7204618	6,283,591	87.22%	114,584	17,361,551	151.52	0.6305%
128.	437,466,552	278,090,175	79.12	3514996	3,107,296	88.40%	90,460	13,781,333	152.35	0.5005%
129.	279,703,340	181,964,130	79.46	2290126	2,020,851	88.24%	73,283	11,532,499	157.37	0.4188%
130.	243,032,462	154,766,003	79.44	1948092	1,703,606	87.45%	64,133	10,067,696	156.98	0.3656%
131.	601,394,804	387,424,626	79.97	4844356	4,222,015	87.15%	102,525	15,656,361	152.71	0.5686%
132.	442,309,704	281,541,907	79.36	3547876	3,133,867	88.33%	90,330	13,709,187	151.77	0.4979%
133.	622,555,718	404,595,387	79.73	5074734	4,441,944	87.53%	112,711	17,010,553	150.92	0.6178%
134.	511,138,174	326,043,727	78.81	4137016	3,627,351	87.68%	93,204	14,546,092	156.07	0.5283%
135.	554,787,344	357,682,079	79.14	4519510	3,982,882	88.13%	96,746	14,604,129	150.95	0.5304%
136.	288,138,456	185,790,940	79.62	2333588	2,017,778	86.47%	74,646	11,527,506	154.43	0.4186%
137.	431,910,542	276,302,250	78.71	3510234	3,095,881	88.20%	89,732	14,109,292	157.24	0.5124%
138.	403,327,542	258,254,557	79.32	3255820	2,851,898	87.59%	90,475	13,915,294	153.80	0.5054%
139.	409,310,176	263,707,086	79.82	3303768	2,926,067	88.57%	89,864	13,971,066	155.47	0.5074%
140.	389,905,046	245,276,474	79.44	3087678	2,730,627	88.44%	79,949	12,444,840	155.66	0.4520%
141.	492,194,412	315,128,702	79.53	3962532	3,473,143	87.65%	96,467	14,643,655	151.80	0.5318%
142.	668,001,072	425,375,209	79.39	5358136	4,703,404	87.78%	108,025	16,654,654	154.17	0.6049%
143.	402,579,132	262,667,356	79.69	3296256	2,832,092	85.92%	85,183	13,005,437	152.68	0.4723%
144.	529,872,664	341,484,784	79.53	4293992	3,767,507	87.74%	99,895	15,631,711	156.48	0.5677%
145.	1,130,412,200	729,297,092	79.53	9170574	7,963,359	86.84%	130,514	19,629,291	150.40	0.7129%
146.	1,106,633,164	710,647,557	79.64	8923332	7,770,259	87.08%	131,456	19,368,163	147.34	0.7034%
147.	868,195,798	552,380,964	79.50	6948296	6,092,040	87.68%	119,048	17,973,250	150.97	0.6527%
148.	1,440,485,432	822,424,673	76.36	10770598	9,499,277	88.20%	133,823	19,760,197	147.66	0.7176%
149.	709,283,206	441,869,862	78.69	5615420	4,904,961	87.35%	94,402	14,049,801	148.83	0.5103%
150.	627,536,230	400,134,945	78.76	5080750	4,466,373	87.91%	103,090	15,956,775	154.78	0.5795%
151.	683,782,120	442,657,825	79.08	5597694	4,901,185	87.56%	108,182	16,579,340	153.25	0.6021%
152.	624,976,486	403,637,570	79.34	5087274	4,482,145	88.11%	103,859	15,875,080	152.85	0.5765%
153.	499,335,516	320,282,146	79.00	4054106	3,570,020	88.06%	94,637	14,707,590	155.41	0.5341%
154.	533,983,768	344,954,436	79.13	4359366	3,843,858	88.17%	95,842	14,502,211	151.31	0.5267%
155.	467,070,056	301,346,100	78.92	3818310	3,281,259	85.93%	89,133	14,000,919	157.08	0.5085%
156.	655,135,894	414,155,645	78.65	5265566	4,490,088	85.27%	105,569	16,253,515	153.96	0.5903%
157.	511,050,304	320,843,953	78.29	4098190	3,366,222	82.14%	90,653	14,038,787	154.86	0.5099%
158.	641,547,152	410,260,975	79.34	5170736	4,491,175	86.86%	121,209	18,225,244	150.36	0.6619%
159.	441,567,556	271,489,951	77.55	3500906	3,061,166	87.44%	87,907	13,586,416	154.55	0.4934%
160.	361,436,782	228,954,031	78.19	2928168	2,545,774	86.94%	82,163	13,009,276	158.33	0.4725%
161.	361,928,248	232,181,623	79.37	2925314	2,583,237	88.31%	82,935	12,768,375	153.96	0.4637%
162.	352,906,322	223,896,193	79.19	2827348	2,479,478	87.70%	77,639	11,993,307	154.48	0.4356%
163.	256,748,262	162,867,184	79.37	2052048	1,791,677	87.31%	66,545	10,184,801	153.05	0.3699%
164.	471,813,016	297,975,710	78.91	3775968	3,204,828	84.87%	89,440	13,540,901	151.40	0.4918%
165.	512,105,956	325,118,057	79.21	4104534	3,610,342	87.96%	93,288	14,283,928	153.12	0.5188%
166.	598,957,270	379,406,240	78.78	4816250	4,251,309	88.27%	102,810	15,543,696	151.19	0.5645%
167.	487,468,420	311,529,420	79.31	3928230	3,425,046	87.19%	88,473	13,466,423	152.21	0.4891%

168.	591,949,486	369,454,526	78.66	4696800	4,016,307	85.51%	102,370	15,284,230	149.30	0.5551%
169.	632,155,970	397,207,302	78.77	5042460	4,439,377	88.04%	104,671	15,748,186	150.45	0.5719%
170.	754,452,426	480,237,193	78.96	6082102	5,367,255	88.25%	105,057	16,043,684	152.71	0.5827%
171.	737,443,420	464,450,908	78.35	5927620	5,225,846	88.16%	110,266	16,721,428	151.65	0.6073%
172.	866,962,184	547,651,195	78.34	6990484	6,130,370	87.70%	119,053	17,842,985	149.87	0.6480%
173.	508,099,286	322,129,625	78.93	4081194	3,536,848	86.66%	93,439	14,078,451	150.67	0.5113%
174.	659,190,640	415,485,413	78.59	5286818	4,648,241	87.92%	105,391	15,944,501	151.29	0.5791%
175.	769,248,118	485,107,053	78.45	6183600	5,435,856	87.91%	111,257	16,681,195	149.93	0.6058%
176.	329,696,926	209,678,004	78.90	2657462	2,320,711	87.33%	74,692	11,312,994	151.46	0.4109%
177.	279,811,006	175,781,114	78.56	2237668	1,974,292	88.23%	68,575	10,619,966	154.87	0.3857%
178.	436,047,906	277,313,489	78.70	3523546	3,017,495	85.64%	85,916	13,021,307	151.56	0.4729%
179.	336,024,374	215,715,159	79.11	2726618	2,406,381	88.26%	78,143	11,861,521	151.79	0.4308%
180.	417,466,532	266,544,640	79.36	3358490	2,958,667	88.10%	85,558	12,907,121	150.86	0.4688%
181.	549,474,138	350,948,926	79.23	4429690	3,836,705	86.61%	95,755	14,391,139	150.29	0.5226%
182.	293,639,522	183,285,017	78.61	2331552	2,043,361	87.64%	67,998	10,265,769	150.97	0.3728%
183.	557,145,088	348,913,350	78.82	4426738	3,896,808	88.03%	96,029	14,281,630	148.72	0.5187%
184.	309,441,376	195,080,801	78.66	2479948	2,195,002	88.51%	74,549	11,211,479	150.39	0.4072%
185.	436,504,830	283,085,132	78.96	3585234	3,162,785	88.22%	89,858	13,399,836	149.12	0.4866%
186.	994,186,228	634,214,395	78.74	8,054,514	7,116,900	88.36%	127,547	18,781,811	147.25	0.6821%
187.	687,671,226	438,463,977	78.59	5,579,318	4,936,913	88.49%	102,918	15,364,641	149.29	0.5580%
188.	577,527,292	365,141,609	78.52	4,650,198	4,072,496	87.58%	99,160	14,772,734	148.98	0.5365%

3 [z] Percent of mapped reads (%) = (No. of mapped reads/Sum of trimmed reads)*100
