# Supplementary material for: Genotyping by Sequencing-Based Discovery of SNP Markers and Construction of Linkage Map from F5 Population of Pepper with Contrasting Powdery Mildew Resistance Trait
Source: Biomed Res Int. 2021 Mar 15;2021:6673010. doi: 10.1155/2021/6673010 (PMC7987414; doi:10.1155/2021/6673010)
Supplement: Supplementary Materials — Supplementary Table 1: the complete GBS statistics of pepper population employed in the present study. Supplementary Table 2: the list of SNP markers discovered and classified into homozygous, heterozygous, and others in all the pepper population employed in this study. Supplementary data 3: the list of genotype data observed for the 188 population using the 1,841 final filtered SNP markers. Supplementary Figure 1: phenotype of the plants used for scoring the disease index in 0-4 scale. [file 6673010.f1.zip › 6673010.f1/Supplementary table 2.pdf]

Supplementary table 2. SNP markers discovered using GBS in the F_5 population of pepper plants along with the parental lines.

Sample	Total	Homozygous (read depth ≥ 90%)	Heterozygous (40% ≤ read depth ≤ 60%)	Homozygous/heterozygous
AR1	81,223	75,784	1,664	3,775
TF68	43,453	38,741	1,481	3,231
1.	33,583	25,932	2,688	4,963
2.	27,444	20,344	2,486	4,614
3.	41,986	32,327	3,448	6,211
4.	43,856	32,516	4,149	7,191
5.	35,994	28,922	2,382	4,690
6.	49,046	33,701	5,925	9,420
7.	39,097	26,714	4,518	7,865
8.	35,128	25,837	3,366	5,925
9.	38,450	28,726	3,461	6,263
10.	36,964	27,614	3,279	6,071
11.	41,121	28,685	4,650	7,786
12.	43,866	28,845	5,642	9,379
13.	55,784	36,935	7,552	11,297
14.	42,685	34,004	3,025	5,656
15.	44,988	30,393	5,417	9,178
16.	63,555	44,861	7,556	11,138
17.	30,522	22,750	2,728	5,044
18.	8,751	7,377	399	975
19.	51,742	39,115	4,803	7,824
20.	29,982	23,069	2,415	4,498
21.	35,794	26,812	3,013	5,969
22.	14,767	12,136	840	1,791
23.	21,271	15,951	1,776	3,544
24.	44,111	29,620	5,453	9,038
25.	55,473	39,653	6,275	9,545
26.	42,447	31,331	3,972	7,144
27.	57,582	42,443	6,305	8,834
28.	53,130	42,456	3,860	6,814
29.	15,278	12,531	873	1,874
30.	23,887	18,559	1,832	3,496
31.	67,510	45,436	9,232	12,842
32.	12,883	9,925	911	2,047
33.	33,887	24,850	3,250	5,787
34.	41,964	28,277	5,215	8,472
35.	42,607	28,858	5,119	8,630

36.	31,580	23,143	2,888	5,549
37.	44,562	34,908	3,641	6,013
38.	26,009	19,462	2,251	4,296
39.	37,361	28,297	3,252	5,812
40.	36,286	27,188	3,233	5,865
41.	20,777	15,628	1,530	3,619
42.	28,608	23,051	1,751	3,806
43.	30,130	22,504	2,675	4,951
44.	10,872	9,139	507	1,226
45.	39,716	28,918	3,942	6,856
46.	38,425	28,295	3,557	6,573
47.	8,603	7,299	401	903
48.	32,426	28,124	1,433	2,869
49.	13,181	10,384	778	2,019
50.	11,993	9,951	590	1,452
51.	29,175	23,387	1,873	3,915
52.	34,372	29,546	1,621	3,205
53.	20,773	15,777	1,583	3,413
54.	65,429	49,900	6,189	9,340
55.	16,663	13,663	1,001	1,999
56.	31,586	22,621	2,989	5,976
57.	39,483	28,807	3,855	6,821
58.	42,413	31,205	4,122	7,086
59.	42,504	30,227	4,524	7,753
60.	12,566	10,722	605	1,239
61.	44,142	32,619	4,157	7,366
62.	23,594	17,903	1,898	3,793
63.	36,183	28,085	2,790	5,308
64.	32,410	24,691	2,616	5,103
65.	20,235	16,289	1,299	2,647
66.	16,821	13,396	1,182	2,243
67.	6,417	5,606	252	559
68.	18,344	14,863	1,200	2,281
69.	12,956	10,796	774	1,386
70.	43,792	32,638	3,966	7,188
71.	20,851	16,169	1,636	3,046
72.	21,037	15,564	1,761	3,712
73.	21,858	17,568	1,312	2,978
74.	22,043	17,475	1,414	3,154
75.	20,551	14,520	1,979	4,052
76.	28,780	22,230	2,284	4,266
77.	9,018	7,854	376	788

78.	27,364	21,355	2,078	3,931
79.	19,598	14,870	1,406	3,322
80.	33,236	24,508	3,094	5,634
81.	40,605	30,266	3,587	6,752
82.	5,673	5,053	208	412
83.	8,356	7,391	365	600
84.	18,427	14,155	1,453	2,819
85.	28,249	19,251	3,130	5,868
86.	16,071	13,504	863	1,704
87.	35,560	25,353	3,525	6,682
88.	38,465	28,644	3,616	6,205
89.	50,441	35,367	5,779	9,295
90.	36,827	25,968	3,860	6,999
91.	43,810	30,665	4,734	8,411
92.	43,100	32,028	3,850	7,222
93.	46,525	35,136	4,046	7,343
94.	28,541	21,558	2,489	4,494
95.	20,565	14,262	2,261	4,042
96.	15,659	10,726	1,672	3,261
97.	32,987	23,193	3,837	5,957
98.	32,698	22,481	3,864	6,353
99.	51,742	36,505	6,794	8,443
100.	32,112	21,757	3,933	6,422
101.	34,316	25,904	3,232	5,180
102.	28,185	20,275	3,050	4,860
103.	26,401	18,280	2,913	5,208
104.	19,805	14,098	2,059	3,648
105.	36,968	29,823	2,741	4,404
106.	28,431	24,742	1,200	2,489
107.	29,095	25,047	1,449	2,599
108.	38,999	23,728	6,324	8,947
109.	38,349	25,898	4,951	7,500
110.	37,704	30,224	2,940	4,540
111.	43,762	29,681	5,852	8,229
112.	28,261	19,316	3,481	5,464
113.	26,936	18,323	3,380	5,233
114.	36,037	23,156	5,242	7,639
115.	43,026	26,458	7,194	9,374
116.	63,583	42,120	10,166	11,297
117.	36,220	23,666	5,050	7,504
118.	36,064	31,509	1,476	3,079
119.	46,231	31,218	6,079	8,934

120.	20,073	14,098	2,067	3,908
121.	31,304	20,903	4,115	6,286
122.	30,021	21,551	3,258	5,212
123.	37,392	32,501	1,717	3,174
124.	34,474	27,818	2,518	4,138
125.	33,008	22,199	4,014	6,795
126.	47,123	32,890	5,866	8,367
127.	39,335	32,420	2,605	4,310
128.	27,069	19,863	2,772	4,434
129.	22,400	15,136	2,592	4,672
130.	20,329	16,473	1,363	2,493
131.	35,339	28,644	2,511	4,184
132.	28,103	20,381	2,895	4,827
133.	37,368	23,421	5,347	8,600
134.	29,204	20,270	3,450	5,484
135.	30,719	19,900	4,132	6,687
136.	23,155	17,336	2,147	3,672
137.	28,973	19,344	3,674	5,955
138.	30,234	20,369	3,713	6,152
139.	26,855	19,679	2,585	4,591
140.	23,536	16,867	2,536	4,133
141.	32,159	21,872	3,957	6,330
142.	39,367	24,567	6,009	8,791
143.	26,656	18,407	3,149	5,100
144.	33,507	22,960	3,942	6,605
145.	48,172	33,761	6,193	8,218
146.	45,171	32,831	4,858	7,482
147.	42,799	29,352	5,517	7,930
148.	47,376	35,057	5,313	7,006
149.	32,288	22,863	3,794	5,631
150.	34,139	25,512	3,252	5,375
151.	36,874	23,854	5,156	7,864
152.	33,305	21,783	4,599	6,923
153.	30,736	20,334	4,024	6,378
154.	29,447	20,014	3,718	5,715
155.	30,610	22,015	3,225	5,370
156.	34,817	21,543	5,293	7,981
157.	30,473	23,081	2,857	4,535
158.	41,735	27,525	5,633	8,577
159.	29,903	21,892	2,999	5,012
160.	27,146	17,926	3,391	5,829
161.	25,810	17,567	2,995	5,248

162.	23,711	17,460	2,382	3,869
163.	20,482	13,123	2,727	4,632
164.	29,915	22,685	2,725	4,505
165.	29,650	20,820	3,350	5,480
166.	33,125	23,744	3,728	5,653
167.	30,407	19,780	4,217	6,410
168.	35,722	22,652	5,134	7,936
169.	35,277	24,957	4,046	6,274
170.	35,666	23,512	4,891	7,263
171.	37,811	25,909	4,782	7,120
172.	41,357	26,444	6,182	8,731
173.	30,790	21,257	3,743	5,790
174.	36,808	23,500	4,416	8,892
175.	36,443	24,861	4,793	6,789
176.	21,574	14,973	2,530	4,071
177.	19,423	13,742	2,032	3,649
178.	27,110	18,766	3,097	5,247
179.	23,586	17,206	2,304	4,076
180.	26,749	19,541	2,673	4,535
181.	33,343	21,428	4,667	7,248
182.	21,099	15,608	2,051	3,440
183.	30,020	20,758	3,710	5,552
184.	22,206	14,450	2,907	4,849
185.	28,097	20,852	2,723	4,522
186.	40,581	28,578	5,086	6,917
187.	34,179	22,966	4,455	6,758
188.	32,716	23,761	3,497	5,458
